# The Incipient Motion Features of Sediment from Yangtze Estuary: Annular Flume Experiments

**DOI:** 10.1038/s41598-017-13651-2

**Published:** 2017-10-16

**Authors:** Huang Wei, Liu Ya-kun, Wu Hua-lin, Wan Yuan-yang

**Affiliations:** 1 School of Hydraulic Engineering, Dalian University of Technology, Dalian, 116024 China; 2 Key Laboratory of Estuarine & Coastal Engineering of Ministry of Transport, Shanghai Estuarine and Coastal Science Research Center, Shanghai, 201201 China

## Abstract

In this study, annular flume experiments are carried out, using the sediment samples collected from Yangtze estuary. The incipient velocity and the incipient shear stress of three different groups of median grain size of sediment are given. The turbulent kinetic energy method (TKE) is used to determine the bed shear stress (τ), by evaluating variations in the suspended sediment concentration (SSC) within the water column. The suspended sediment concentration increases with the increase of the bed shear stress. When the sediment concentration reaches a certain concentration, the rate of change in τ obviously slows down. As the concentration increasing again, the bed shear stress grows rapidly with different growth rate attributed to different grain size. The results of the experiments indicate that SSC and grain size have strong influence on τ.

## Introduction

The dynamics of sediment transport, including incipient velocities, erosion, settling velocities and consolidation, etc., has remained a focal issue in scientific research and engineering practice^1–4^. The bed shear stress, which plays a dominant role in the fine sediment erosion, settling and transport of sediments, is often derived from measured velocity profiles on the basis of the Kármán-Prandtl model^5,6^. However, in flow with suspended sediment, this method would overestimate the bed shear stress. Previous studies have demonstrated the drag reduction effect in flume experiments, *in-situ* experiments, and *in-situ* observations^7–13^. Gust found that the true bed shear velocity was reduced by 40% under the conditions of his experiments. Li and Gust also found that the directly measured shear velocity was  reduced by 70% relative to the profile-derived shear velocity in the logarithmic layer. Amos found that the shear velocity dropped 5–10% as the mud concentration increased from 0 to 200mgl^−1^ for a constant mean velocity. Amos *et al*. proposed a relationship between drag reduction and clay concentration. The above review of previous work leads to a same conclusion that the SSC has a strong influence on bed shear velocity. Annular flumes are ideal experimental devices for the study of coastal and estuarine fine-grained sediment dynamics in both laboratory and field applications. An annular flume is a typical ring shaped flume with two rotating elements, the top lid and the flume, which can be rotated independently. A uniform tangential flow velocity is generated by rotating the top-lid and the flume in opposite directions. The main advantage of using an annular flume for sediment experiments is the infinite flow length. There is no inlet or outlet and no pumps are used so that the flocculation and bed form would not be destroyed.

In this contribution, a series of annular flume experiments were carried out in Shanghai Estuarine and Coastal Science Research Center. The sediment samples and water were collected form the Yangtze estuary. This paper gives the corresponding incipient velocities and the critical bed shear stresses for the different sediment size groups. And the incipient motion features of sediment for different sediment size groups were discussed.

### Experimental conditions

#### Bed material

**Figure 1:**
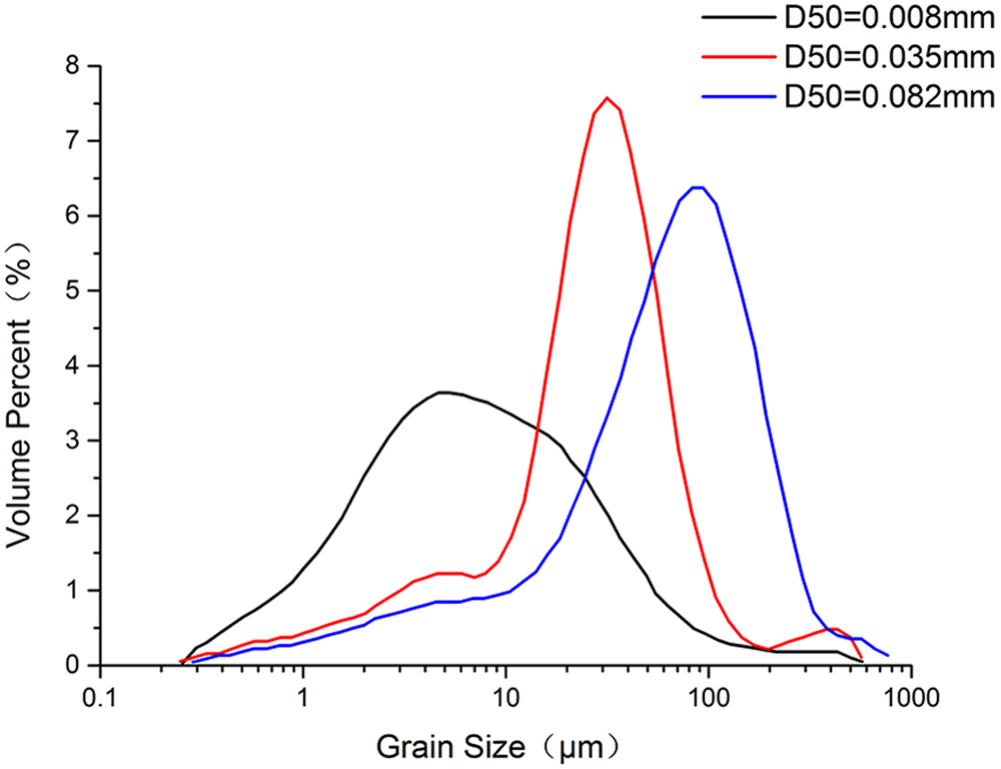
The grain size distribution curves of three groups of sediment.

Three different groups of median grain size of sediment samples and the test water were collected from the North Passage in Yangtze estuary for the flume experiments. The samples was measured by MS2000 particle size analyzer. The median grain size of three groups of sediment samples were 0.082mm, 0.035mm and 0.008mm respectively, the corresponding grain size distribution curves are shown in Fig. 1. They were redefined to three types: coarse particle, medium particle and fine particle. The fine particle was clay-dominated, the medium particle and the coarse particle were silt-dominated. And they were all clay-silt-sand mix and cohesion-dominated sediments. The initial thickness of the bed material in the flume was set at 2.0 cm and water depth was set at 15.0 cm. The seawater salinity(PSU) was 9‰ and the temperature was ranged from 13.5 °C to 15.6 °C. This temperature fluctuation was affected by the environment, because each set of the experiments lasted about 30 hours.

#### Annular flume

The experiments were carried out in the annular flume^14^ made by Shanghai Estuarine and Coastal Science Research Center. The flume consists of two 50 cm high acrylic cylinders with inner and outer diameters of 184 cm and 216 cm, respectively, and the flume section is 16 cm wide. The lid consists of a rotating ring, which is height-adjustable to control the water depth. The lid and the flume are driven by separate step-less motors in order to facilitate rotation in opposite directions and thereby generate shear stresses and currents. The occurrence of secondary radial flows is minimized by controlling the ratio of the rotating speed of the lid to that of the flume^15^. When the secondary flow is minimized and the main current reaches steady flow conditions, the depth-averaged flow velocity can be calibrated against the fixed rotating speeds of both the lid and the flume. The optimal rotating speed rate of this annular flume is about 3.0 which was concluded by a series of experiments with the seawater at a height of 15 cm.

### Experimental setup

#### Stage.1

The initial thickness of the bed material in the flume is set at 2.0 cm. The seawater depth is set at 15.0 cm.

#### Stage.2

Launch the annular flume to increase the flow speed until all the sediment go into the seawater. Then turn off the flume to make the sediment settle naturally. When the thickness of the bed has no obvious changes (The settling time was about 2 days), the next stage can be proceeded.

#### Stage.3

**Figure 2:**
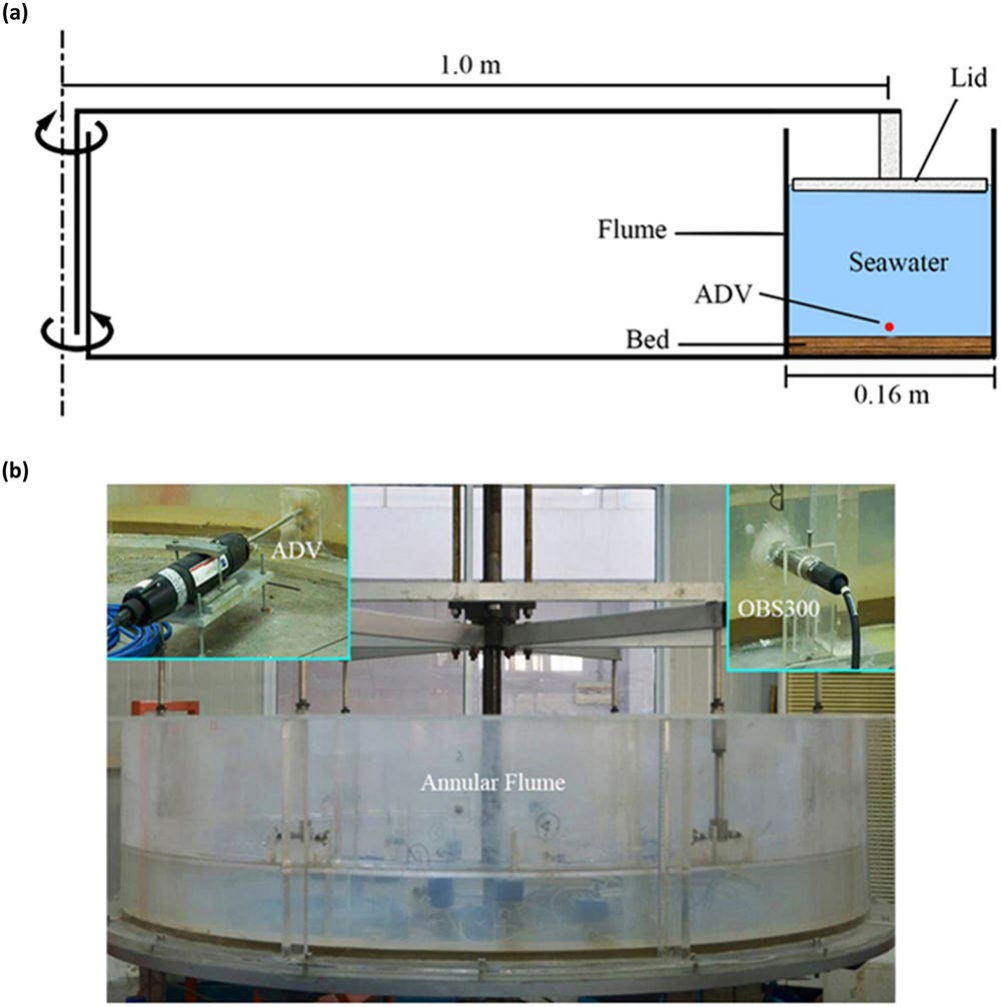
(**a**) Schematic drawing of the annular flume. (**b**) Experimental equipment.

Launch the annular flume and control the flow velocity from low speed to high speed, each velocity level remained 2–4 hours. The speed monitoring point is set in the middle of the section and 0.2 cm high from the bed material as shown in Fig. 2. The velocities are collected by ADV (Range: ± 0.0 – ± 4 m/s, Accuracy: ± 0.5% of measured value ± 1 mm/s), thus the mean velocities and turbulent velocity fluctuations can be obtained.

#### Stage.4

The turbidity is collected by turbidity meter (OBS300). From the OBS variable curve, it is easy to see when the suspended sediment concentration is stable. Then collect the water samples from the top, middle and bottom layers in the flume (4 cm, 8 cm and 14 cm high from the bottom). The SSC of each sample is obtained by oven drying method.

#### Stage.5

After the samples collection, increase the current velocity and observe the changing curves of velocity and turbidity until they are stable, then repeat stage 4 and stage 5. The experiment is over when all the bed material go into the seawater, which means the turbidity doesn’t increase while the current velocity increased.

### Data processing

The calculation of the bed shear stress (τ)^16^ is based on turbulent kinetic energy method(TKE)^17^,

1 $$E=\frac{1}{2}({u^{\prime} }^{2}+{v^{\prime} }^{2}+w{\text{'}}^{2})$$

2 $${\rm{\tau }}=0.19\rho E$$

where *u*′,*v*′,*w*′ are the turbulent velocities, *ρ* is the mass density of water (including suspended load), which can be estimated by Eq. (3)^18^:

3 $$\rho =1000+0.62C$$

where *C* is the depth-averaged value of SSC of samples which were collected from the top, middle and bottom layers in the flume.

## Results and Discussion

### The incipient velocity and critical shear stress

**Figure 3:**
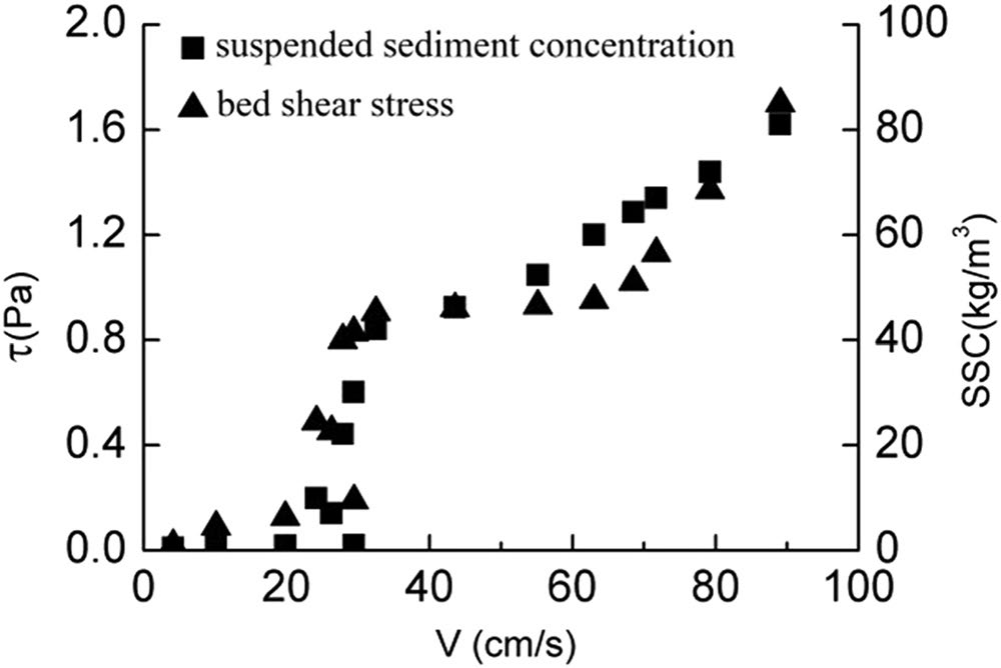
The relationship between coarse sediment’s bed shear stress, velocity and suspended sediment concentration.

**Figure 4:**
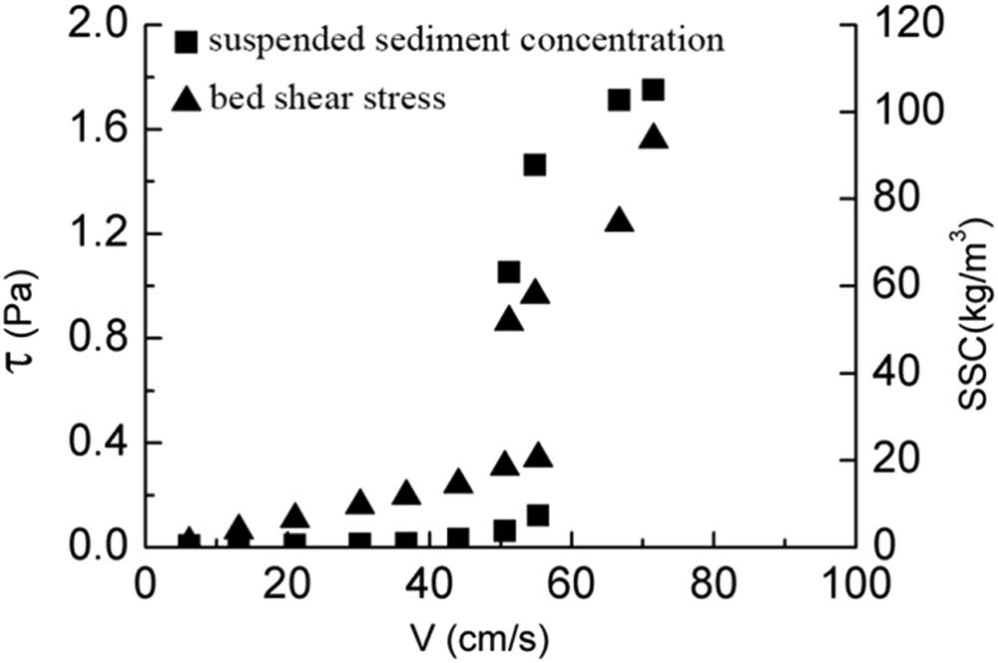
The relationship between medium sediment’s bed shear stress, velocity and suspended sediment concentration.

**Figure 5:**
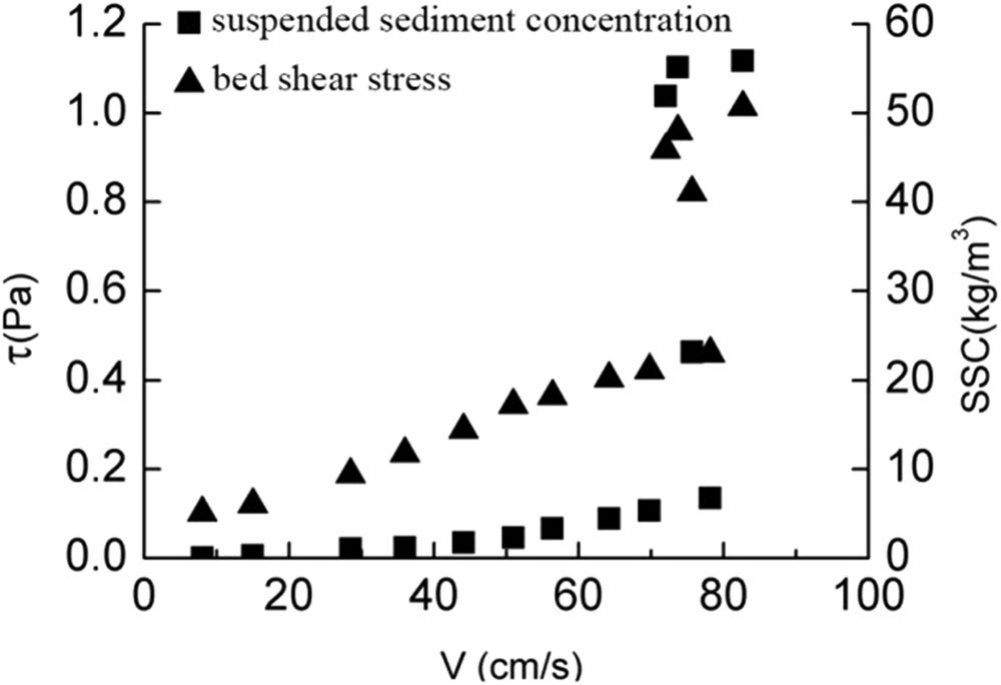
The relationship between fine sediment’s bed shear stress, velocity and of suspended sediment concentration.

As it is shown in Fig. 3, the bed shear stress increases slowly with the increase of SSC and velocity before the coarse sediment goes into incipient motion. When the bed shear stress increases to a certain level (critical shear stress), the coarse sediment start moving and the suspended sediment concentration grows. Meanwhile the bed shear stress increases rapidly which accelerates the suspended sediment concentration’s growth. The incipient velocity and critical shear stress can be known from the Fig. 3: *V* = 0.29 m/s, τ_*cr*_ = 0.19 Pa. Similarly in Figs 4 and 5, the incipient velocity and critical shear stress can be seen: *V* = 0.55 m/s, τ_*cr*_ = 0.34 Pa for the medium sediment and *V* = 0.78 m/s,τ_*cr*_ = 0.46 Pa for the fine sediment. As we known, the classical Shields curve described the relation between Shields number and Reynolds number of sediment^19^,

4 $${\theta }_{c}=\frac{{\tau }_{c}}{({\gamma }_{s}-\gamma )}=f({R}_{{e}_{\ast }})=f(\frac{{u}_{\ast }D}{v})$$

From the Shields curve, when the Reynolds number of sediment is close to 10, the bed material size is close to the near-wall thickness and the sediment is easy to move. The median sizes of three groups in this experiment were all less than 0.1mm, and the Reynolds numbers of sediment are less than 10, So the smaller the particle size is, the greater the critical shear stress is. This was due to the gain size distribution. Most particles belonged to clay. The particles smaller than 0.1mm were cohesion-dominated. So the smaller the sediment is, the more shear stress is needed to break the bonding force between particles, which means the higher the incipient velocity is.

### The bed shear stress in the incipient motion

**Figure 6:**
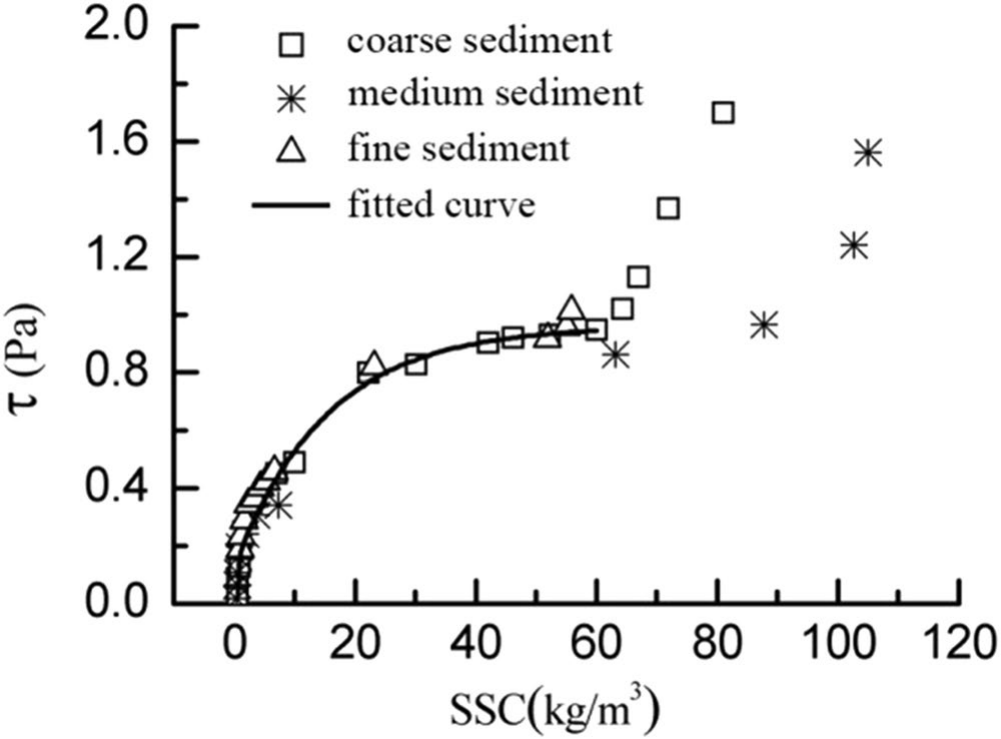
The relationship of three different sediment size groups between the bed shear stress and suspended sediment concentration.

It can be seen from Figs 3–5, the bed shear stress and the SSCs increase linearly with the increasing velocity before the sediment start moving. Once the velocity reaches the incipient velocity, the sediment start moving into the seawater. As the suspended sediment concentration grows up, the bed shear stress increases rapidly to a high level (almost twice of the critical shear stress). So the influence of the rapid growth of the SSC on the bed shear stress cannot be ignored. The relationship of three different sediment size groups between the bed shear stress and SSC is plotted in Fig. 6. The figure shows that, as the flow velocity increases, the relationship between τ and SSC follows a logarithmic growth trend, and the bed shear stress growth trend of three groups with the increasing SSC is consistent before the SSC reaches 60 kg/m^3^. When the SSC grows up over, the growth rate of the bed shear stress of coarse sediment is faster than that of medium sediment. Because the maximum SSC of the fine sediment is 58 kg/m^3^, the higher SSC datas are lacked as shown in Fig. 6.

## Conclusions

In this paper, using annular flume experimental study on incipient motion, fine sediment in the Yangtze River estuary as samples come to a conclusion as follows:

1. It is concluded that in the Yangtze estuary three different values of sediment starting velocity and critical stress are obtained, the median particle size of 0.082 mm, 0.035 mm and 0.008 mm of incipient motion velocity are 0.29 m/s, 0.55 m/s and 0.29 m/s, respectively, the critical shear stress are 0.19 Pa, 0.34 Pa and 0.46 Pa. Thus, the median size less than 0.1 mm of fine sediment, the critical shear stress and starting velocity decrease with the increase of particle size, the particle size is larger, the more likely it is easy to start. This is due to bonding force between particles. This test of incipient velocity and critical shear stress provides a reliable basis for the Yangtze River estuary numerical simulation parameters selection.
2. Before the sediment starting incipient motion, the bottom shear stress increases with the increasing flow velocity (logarithmic growth) and the bottom shear stress grows slowly, when the concentration reaches 60 kg/m^3^, the bottom shear stress is growing rapidly, but for the different particle size of sediment at the bottom, the shear stress is not the same growth rate. In these tests, the shear stress at the bottom of the coarse particle growth has grown faster than the medium particles. It is known that not only the SSC, but also bed particle size affects bed shear stresses. Therefore, the results in this contribution confirm that effect.
