# Author Correction: The Incipient Motion Features of Sediment from Yangtze Estuary: Annular Flume Experiments

**DOI:** 10.1038/s41598-018-24097-5

**Published:** 2018-04-12

**Authors:** Huang Wei, Liu Ya-kun, Wu Hua-lin, Wan Yuan-yang

**Affiliations:** 1 School of Hydraulic Engineering, Dalian University of Technology, Dalian, 116024 China; 2 Key Laboratory of Estuarine & Coastal Engineering of Ministry of Transport, Shanghai Estuarine and Coastal Science Research Center, Shanghai, 201201 China

Correction to: *Scientific Reports* 10.1038/s41598-017-13651-2, published online 16 October 2017

The Acknowledgements section in this Article was omitted. The Acknowledgements section should read:

“This work was supported by the National Natural Science Foundation of China (No. 51479022).”

